# “Hearing faces and seeing voices”: Amodal coding of person identity in the human brain

**DOI:** 10.1038/srep37494

**Published:** 2016-11-24

**Authors:** Bashar Awwad Shiekh Hasan, Mitchell Valdes-Sosa, Joachim Gross, Pascal Belin

**Affiliations:** 1 Centre for Cognitive Neuroimaging, Institute of Neuroscience and Psychology, University of Glasgow, Glasgow, United Kingdom; 2 Institute of Neuroscience, Newcastle University, Newcastle, United Kingdom; 3 Cuban Center for Neuroscience, La Habana, Cuba; 4 Département de Psychologie, Université de Montréal, Montréal, Québec, Canada; 5 Institut de Neurosciecnes de la Timone, UMR 7289, CNRS and Aix-Marseille Université, Marseille, France

## Abstract

Recognizing familiar individuals is achieved by the brain by combining cues from several sensory modalities, including the face of a person and her voice. Here we used functional magnetic resonance (fMRI) and a whole-brain, searchlight multi-voxel pattern analysis (MVPA) to search for areas in which local fMRI patterns could result in identity classification as a function of sensory modality. We found several areas supporting face or voice stimulus classification based on fMRI responses, consistent with previous reports; the classification maps overlapped across modalities in a single area of right posterior superior temporal sulcus (pSTS). Remarkably, we also found several cortical areas, mostly located along the middle temporal gyrus, in which local fMRI patterns resulted in identity “cross-classification”: vocal identity could be classified based on fMRI responses to the faces, or the reverse, or both. These findings are suggestive of a series of cortical identity representations increasingly abstracted from the input modality.

- Local patterns of cerebral activity measured with fMRI can classify familiar faces or voices.
- Overlap of face- and voice-classifying areas in right posterior STS.
- Cross-classification of facial and vocal identity in several temporal lobe areas.

The ability to recognize familiar individuals is of high importance in our social interactions. The human brain achieves this by making use of cues from several sensory modalities, including visual signals from the face of a person and auditory signals from her voice^1^,^2^. There is evidence that these cues are combined across senses to yield more accurate, more robust representations of person identity—a clear case of multisensory integration^3^,^4^,^5^. For instance, familiar speaker recognition is faster and more accurate when the voice is paired with a time-synchronized face from the same individual than when presented alone, and slower and less accurate when paired with the face of a different individual^3^.

The contribution of different sensory modalities to person perception is acknowledged in particular by cognitive models such as Bruce and Young (1986)’s model of face perception. Specifically they proposed the notion of “person identity nodes” (PINs): a portion of associative memory holding identity-specific semantic codes that can be accessed via the face, the voice or other modalities: it is the point at which *person* recognition, as opposed to face recognition, is achieved^6^,^7^.

Whether the PINs have a neuronal counterpart in the human brain remains unclear, in part owing to the fact that most studies of person recognition—either using neuropsychological assessment of patients with brain lesions, or neuroimaging techniques such as functional magnetic resonance imaging (fMRI) in healthy volunteers—have focused on single modality, mostly face, then, far second, voice; only few studies have investigated the cerebral bases of person recognition based on more than one sensory modality^1^,^4^.

Lesion and neuroimaging studies have suggested potential candidate cortical areas for the PINs, including the precuneus^8^, parietal and hippocampal regions^9^,^10^,^11^, posterior superior temporal sulcus (pSTS)^12^,^13^ or the anterior temporal lobes^14^. However a PIN, as defined in Bruce & Young (1986), would correspond to a patient with a brain lesion preserving recognition and feeling of familiarity based on single modalities separately but who could not retrieve semantic information on the person, and not associate the face and voice of the person; such a patient has not yet been identified^1^. Other studies suggest that amodal representations could rather emerge from cross-talk interactions between modality-specific areas^1^: voice and face-sensitive areas are not only connected via direct anatomical projections^15^ but also functionally connected during familiar voice recognition^16^.

Multi-voxel pattern analyses (MVPA) offer a powerful means of extracting information contained in distributed fMRI activity^17^,^18^: their enhanced sensitivity compared to classical univariate fMRI analyses has contributed to clarifying the neural correlates of unimodal face^19^,^20^,^21^,^22^,^23^ or voice^24^,^25^ identity processing. To our knowledge MVPA have not been used yet to examine the cerebral bases of multimodal person identification although they have provided exciting insights in the integration of emotional information across senses. Peelen *et al*.^26^ used a searchlight information-based analysis^27^ to compare the fMRI activation patterns elicited in local spherical clusters of voxels by emotional stimuli presented in three modalities: faces, voices and bodies. They found two clusters across the whole brain, located in rostral medial prefrontal cortex and in the left pSTS, in which patterns of activity associated with the same emotions from different modalities were more similar to each other than patterns associated with different emotions, strong evidence for supramodal representations of emotion^26^.

Here we used fMRI to measure blood oxygenation level dependent (BOLD) signal as an indirect index of neuronal activity in normal volunteers engaged in an identity recognition task. They were performing a 4-alternative forced choice classification of the identity of four familiar persons recorded while saying the word “had” either as a video combining the face and the voice (Face-Voice condition), a silent video of their face (Face condition) or the audio recording of the voice (Voice condition). We used a slow-event related design to maximize independence between trials and a large number of trials to enable robust subject-level analyses. Searchlight MVPA was used across the whole grey matter mask of each subject to localize brain areas in which local patterns of cortical activity could be used to train a support vector machine (SVM) classifier to classify the identity of the familiar persons viewed and/or heard. We asked whether some cortical areas could result in identity classification across sensory modalities.

## Results

**Figure 1:**
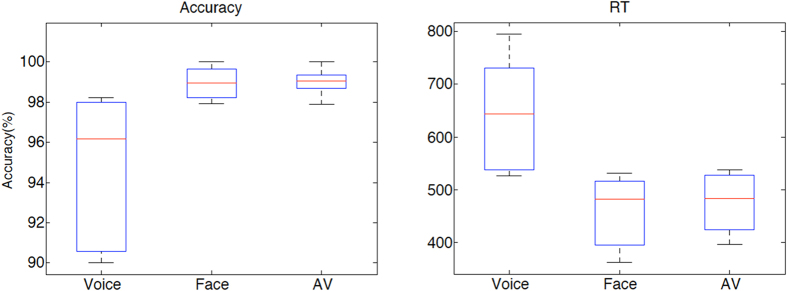
Behavioural results. Distribution of average accuracy and reaction time (in ms) at the identity classification task across the five participants. AV: audiovisual Face-Voice condition.

All five participants performed the identity classification task with near-ceiling accuracy during scanning (Fig. 1). Individual one-way ANOVAs showed that average percent correct accuracy was lower and average reaction time (RT) longer in the Voice condition (p < 0.05 two-tailed; Accuracy: mean = 94.6%, range across the five subjects = [90.0–98.2]; RT: mean = 643 ms, range of mean = [542–795]) than in the Face (Accuracy: mean = 98.9%, range = [97.9–100]; RT: mean = 458 ms, range = [362–531]) or the Face-Voice (Accuracy: mean = 99.0%, range = [97.9–100]; RT: mean = 475 ms, range = [396–537]) conditions (differences significant in 4/5 participants).

Univariate fMRI analysis confirmed the involvement of visual and auditory cortex in stimulus processing, revealing overlapping activation of bilateral fusiform cortex and medial prefrontal cortex in the Face and the Face-Voice conditions, and of bilateral superior temporal gyrus and sulcus in the Voice and the Face-Voice conditions. No significant activity differences between the four identities were observed in any part of the brain for any stimulation conditions (all q > 0.01 FDR) with the univariate analysis.

**Figure 2:**
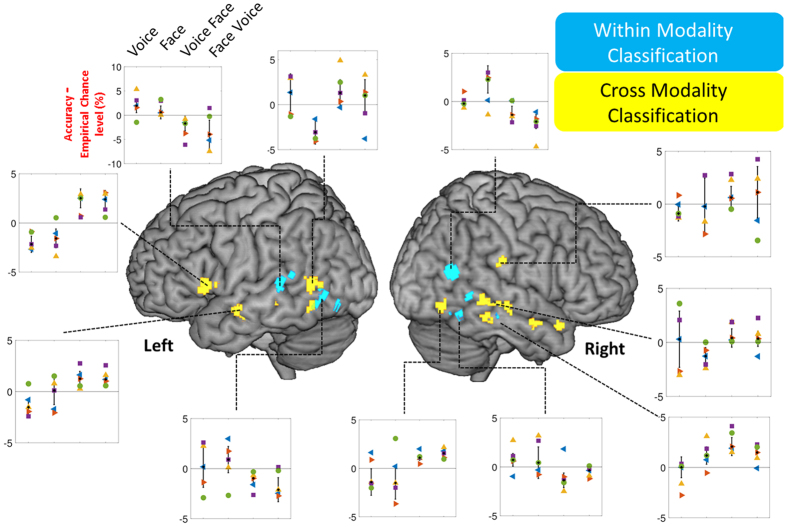
Identity cross-classification in multiple cortical areas. Voxels overlaid in color on surface rendering of the left and right hemispheres are the centers of spherical ROIs resulting in significantly (q < 0.01 FDR corrected) above-chance classification of the stimulation conditions in at least 4 of the 5 subjects. Blue voxels: above-chance within-modality classification (train on voice and test on voice; train on face and test on face); Yellow voxels: above-chance cross-modal classification (train on voice and test on face; train on face and test on voice). Panels show for selected clusters the individual values (coloured shapes: Subjects 1–5) of the cluster-average difference between classification accuracy and the empirical chance level (determined via permutations for each voxel) for each of the four classification schemes. Bars indicate standard deviation across the group mean. Voice: train and test on voice; Face: Train and test on face; Voice-Face: train on voice and test on face; Face-Voice: train on face and test on voice.

In the MVPA analysis (cf. Methods) all participants individually showed areas of cortex in which local patterns of fMRI activity resulted in significantly (0.01 FDR corrected) above-chance stimulus classification, either the voice or the face. The coincidence map of Fig. 2, which shows in the standard stereotactic space of the Montreal Neurological Institute (MNI) the voxels present in the individual classification accuracy maps of 4 or 5 of the 5 participants, reveals several areas consistently involved across individuals. For faces, classifying areas included left fusiform gyrus, right inferior temporal gyrus and right posterior STS. For voices, classifying areas were located along left occipital cortex, left mid STS, right mid STS/middle temporal gyrus and posterior STS (Fig. 2, Table 1).

Next we probed the modality-dependence of these local representations of face and voice stimuli in two different, complementary ways. First we asked whether any overlap in the stimulus-classifying areas based on face or voice could be observed. Only a single region of cortex showed such overlap consistently across participants: spherical ROIs centered on right posterior STS (pSTS) could classify both faces and voices (Fig. 2, Table 1).

Second, we asked whether some areas could result in *identity cross-classification*, that is, whether they could classify test fMRI volumes measured in response to presentation of the familiar identities in one modality (e.g., the face) after training the SVM classifier based on fMRI volumes measured in response to the other modality (e.g., the voice). All participant showed such areas resulting in cross-classification of identity either in one direction (train classifier on voices, test on faces) or the other (train on faces, test on voices) or both (Figure S1). The coincidence map of these regions across the five participants (Fig. 2) revealed a number of these identity cross-classifying areas consistently located across individuals: most consisted of small patches of cortex located in the temporal lobe along the whole antero-posterior extent of the STS bilaterally; additional cross-classifying areas were found in left inferior prefrontal cortex and in right Rolandic operculum. Remarkably, several of these cross-modally identity classifying areas showed an overlap of the two directions of cross-classification: fMRI patterns measured in these areas resulted in above-chance cross-classification of identity in both directions, i.e., from face to voice and from voice to face (Table 1).

## Discussion

We used an fMRI searchlight MVPA approach to investigate the cerebral processing of identity information from the auditory and visual sensory modalities. We searched the whole brain of healthy participants for areas in which local fMRI patterns measured in response to the face or voice of four familiar individuals contained sufficient information to classify the face or voice. All participants individually showed areas resulting in significantly above-chance classification of face or voice stimuli based on the responses of local voxel clusters, consistent with previous results. We also found, for the first time, local areas resulting in identity cross-classification, that is, successful classification of identities in one modality based on fMRI responses in the other modality. We argue these results reinforce the notion of amodal representations of person identity in the human brain.

We found that the face and voice of familiar persons can be decoded within-modality based on local fMRI patterns. All five participants showed areas in which fMRI patterns measured in 8 mm-radius spheres in response to the face or voice of the four familiar persons resulted in significantly (q < 0.01 FDR corrected) above-chance classification of face or voice by a classifier trained on stimuli in the same modality: face classification based on fMRI responses to the faces; voice classification based on fMRI responses to the voices (Fig. 2, Table 1).

Face stimulus classification could be performed based on the local fMRI signal in regions of fusiform and occipital cortex (Fig. 2, Table 1). This result is consistent with previous reports of face identity classification based on local fMRI clusters in fusiform areas^19^,^20^,^21^,^22^ suggesting that activity in these areas of the core face processing network already contain sufficient identity information to discriminate between facial identities. We did not observe face classification in more anterior areas of the anterior temporal lobe reported by some studies^19^,^20^,^23^.

Voice classification based on local fMRI clusters is, to our knowledge, a novel result. Previous reports have demonstrated classification of speaker identity based on fMRI responses to voices^24^,^25^ but this classification was based on large number of voxels non-continuously drawn from extended areas of cortex. In Formisano *et al*.^24^’s pioneering study all voxels in auditory cortex were initially considered for training and testing the SVM classifiers, then uninformative voxels were progressively eliminated (“recursive feature elimination”^28^). This procedure resulted in maps of voxels spanning discontinuously the whole auditory cortex and that collectively resulted in above-chance classification of speaker’s voice or vowel^24^. These findings have been interpreted as evidence for a distributed coding of speaker identity, where information on speaker identity is possibly represented across multiple cortical areas. Here we show that significant voice classification is possible based on much smaller sets of voxels, suggesting that cortical representations of speaker identity in the temporal lobe are not necessarily distributed over large cortical areas. Thus speaker recognition could involve both local as well as distributed representations depending in particular on the relevant stimulus features.

Note that the above results should be interpreted with caution: the face (or voice) classification observed here does not necessarily imply face (or voice) *identity* classification. Previous studies having examined identity classification have used multiple stimuli for each identity (e.g., faces viewed from different viewpoints; speakers pronouncing different words) such that the classifier had to generalize over several different instances of the identity—in effect solving the invariance problem our brain is confronted with when assessing identity^19^,^20^,^21^,^22^,^24^,^25^. Here we only used a single stimulus per modality per identity, such that accurate face or voice classification by a classifier does not imply that this classifier could generalize to other stimuli from the same identity. We tested for low-level stimulus classification, not higher-level *identity* classification. Of course, this limitation disappears when training and testing sets correspond to stimuli from different modalities (cf. below).

The first clue towards multi-modality in cortical representations of identity comes from the overlap of the (within-modality) face and voice classification maps. All participants showed such areas of overlap of face and voice classification in which local fMRI clusters resulted in above-chance classification for both faces and voices with right pSTS being the only region consistently located across participants (Fig. 2). This result is consistent with a growing body of evidence on the multi-modal nature of stimulus representations in pSTS^29^,^30^,^31^,^32^. Watson *et al*.^12^ found that areas in pSTS show comparable BOLD signal increases compared to baseline when participants were stimulated with faces or voices^12^. Right pSTS was also highlighted in the first report of cross-modal adaptation in processing facial and vocal expressions of emotion: activity in this area measured in response to emotional face-voice was greater when the emotion expressed by the face of a stimulus was different from the emotion expressed by the voice of previous stimulus and smaller when the facial and vocal emotions were similar^33^. Together, these results highlight right pSTS as an important region of convergence of the visual and auditory streams of person-related information processing.

The most novel result of the present study is our observation of cross-modal classification of identity in several areas of cortex. We show for the first time that training SVM classifiers with BOLD signal read from local clusters of fMRI voxels in response to identities presented in one modality can successfully decode the same identities presented in the other modality.

Most such cross-classifying areas were found along the STS/MTG bilaterally, consistent with the notion of a stream of person-related information processing directed towards anterior temporal lobe^2^. Unexpectedly right pSTS does not appear in the group-level map. Cross-classifying clusters are found in the right pSTS at the individual level (in all subjects except P3, cf. Figure S1) but less consistently located across subjects than clusters located more inferiorly and anteriorly and so not appearing on the coincidence map. Why the anterior temporal poles were not observed in our results, despite their likely involvement in multimodal/amodal representations of individuals^1^, remains unclear. This is unlikely due to signal-to-noise ratio differences in anterior compared to posterior temporal regions^22^ as we found above-chance classification for other classification schemes in some quite anterior temporal lobe clusters (Fig. 2). We also observed identity cross-classification in left inferior prefrontal cortex. Although this region is not typically part of identity-processing areas, it could reflect the local processing of linguistic information accessible from both face and voice modalities: the persons’ names. Indeed, several aspects of the stimuli and task performed by the participants other than identity processing could account for some of the results observed, including covert naming of the familiar persons viewed or heard, as well as recall of semantic information and episodic memories associated with these persons. It is probably those aspects of the experimental task that are reflected by the involvement of the inferior frontal gyrus and supra-marginal gyrus (Table 1).

Importantly, the limitations of a single stimulus per identity per modality discussed above do not bear on the interpretation of the cross-modal results: the training and test sets of fMRI data were acquired not just on different stimuli from the same identities, as has been performed by other ‘unimodal’ studies^19^,^20^,^21^,^22^,^24^,^25^ but on different stimuli in different sensory modalities – arguably a more complex generalization problem than that posed by different person-related stimuli in the same modality^26^. So the cross-classification observed here is a ‘true’ identity classification, as opposed to the *stimulus* classification of our within-modality results.

This evidence, combined with previous results, provides crucial novel insight into the nature of the representation of person-related information in the human brain. The format of person representation in some cortical areas appears abstracted enough from the input modality as to allow correspondence between different modalities. In some areas cross-classification only worked in a single direction, but most of these cross-classifying cluster combined slightly different regions that cross-classified in the two opposite directions (face to voice, and voice to face).

These results are suggestive of a hierarchy of representations of person-related information with increasingly abstract, modality-free representations. Person representations starting in unimodal areas of visual and auditory cortex would be tied to a sensory modality, possibly allowing classification of different stimuli from a same identity but only in that modality. These representations would be characterized by a first point of convergence in right pSTS, in which the representations in the different modalities would start to become merged, possibly via an increasing proportion of multimodal neurons^33^ in this area located at the junction of auditory and visual processing streams. These representations would then become increasingly abstracted from the input modality along a rostro-caudal stream along STS/middle temporal gyrus possibly directed towards the anterior temporal lobes^2^: such modality-free, or amodal, representations of identity would in particular be able to support cross-classification of identity.

Overall, our results are consistent with a distributed cerebral PIN, implemented as a series of representations increasingly abstracted from the input modality ranging from unimodal (in visual and auditory cortex), to multi-modal (in right pSTS), to amodal, possibly oriented along STS/MTG. The recognition of individuals based on their face and voice would flexibly emerge from the complex, multidirectional interactions between these different representations as a function of information conveyed by each modality, context, etc. Future studies combining large-scale neuroimaging techniques with local recordings and/or perturbation of single-cell or population activity in animal models, including careful examination of effective connectivity between the different areas, will be key to precise the exact mechanisms underlying such hierarchy at the neuronal level^2^.

## Methods

### Participants

Five healthy volunteers participated in this study (Four females and one male, mean age ± s.d.: 26.8 ± 2.4 years). All participants were members of the Institute of Neuroscience and Psychology at Glasgow University. They had self-reported correct hearing and vision with no history of any hearing, visual or neurological conditions. The ethics committee at the Institute of Neuroscience and Psychology approved the study which was conducted in accordance with the guidelines from the British Psychological Society. Participants provided written informed consent and were compensated £6 per hour for their time.

### Stimuli

Four speakers were recorded for this study (two males and two females) in a sound proof room under standard studio lighting condition. Speakers faced the camera directly and at a distance sufficient to show the whole face in the frame. Videos were recorded at a 25 frames/sec rate with a resolution of 640 × 480 pixels using a HD Sony camcorder. Audio was recorded at a sampling rate of 48 kHz and a 16 bits per sample resolution using an M300 condenser microphone (Microtech Gefell GmbH, Germany). All speakers were members of the Institute and familiar to all the participants via daily interaction over the course of several months or years. The speakers were recorded uttering the syllable “had”. Only one recording per speaker was used in the experiment. iMovie (Apple Inc.) was used to crop the stimuli to 400 with average delay between face and voice onset at 52 ± 19 ms (face leading voice). As a result three versions of the stimuli were produced: I) Voice: only the audio is presented. II) Face: only the silent video is presented. III) Face-Voice: the audio-visual stimulus is presented without modification.

Stimuli were presented in the scanner using Media Control Functions (DigiVox, Montreal, Canada) via electrostatic headphones (NordicNeuroLab, Norway). Sound pressure level was set at 80 dB SPL(C) as measured using a Lutron Sl-4010 sound level meter. Once the participants were installed in the scanner they were presented with sound samples to verify that the sound pressure level was balanced and loud enough to compensate for the scanner noise.

### Experiment Design and Task

Each identity (n = 4) was presented in the three different conditions: Voice, Face, and Face-Voice, resulting in 12 conditions in total. Participants were presented with a stimulus corresponding to one of the four identities in a randomized order, and participants were instructed to classify the identity of the stimulus by pressing one of four buttons with the right hand. No feedback was given to the participant. The experiment was controlled using Matlab (The Mathworks, Inc, USA) and Psychtoolbox^34^. Participants were given time to practice the experiment and the mapping between buttons and identities before entering the scanner.

Experimental design followed a slow event-related paradigm to minimize contamination of consecutive hemodynamic responses, and hence minimize any potential cross talk between BOLD responses to the V and F stimuli. Inter-stimulus interval was set randomly between 10 and 18 seconds with the onset of the stimuli locked to BOLD volume acquisition.

Data were recorded from each participant over several sessions each containing 6–8 blocks (number of total blocks per participant (mean ± s.d.): 29.2 ± 5.4). Each block lasted around 8 minutes with each condition presented twice. Two-minute breaks were given between each two consecutive blocks, during which the participants were instructed not to move their head. Overall 700.8 ± 129.7 trials were presented corresponding to the 4 identities and three modalities for each participant.

Participants were instructed to perform a 4-alternative forced choice identity classification task using 4 buttons of an MR compatible response pad (NordicNeuroLab, Norway). Reaction times (relative to sound onset for V condition and face onset for F and FV conditions) were collected within a response window limited to two seconds after stimulus onset.

### fMRI Data Acquisition

Scanning was carried out in the Centre for Cognitive Neuroimaging at the University of Glasgow. A 3-Tesla Siemens (Erlangen, Germany) TIM Trio scanner was used with a 32-channel head coil.

#### Functional Scans

High spatial resolution (2 × 2 × 2 mm3) functional images of the whole brain were collected. A relatively long TR was used (TR = 3.6 s) to ensure full coverage of the brain. Echo time (TE) = 39 ms, flip angle = 82 degrees, slices = 51 × 2 mm thickness × 10% gap, field of view (FOV) = 192 mm, matrix = 96 × 96, iPAT acceleration factor = 2, maximum number of volumes = 140, and maximum acquisition time (TA) = 8 min 36 seconds. The first 10 seconds of the functional run consisted of “dummy” gradient and radio frequency pulses to allow for steady state magnetization during which no stimuli were presented and no fMRI data collected.

#### Anatomical Scans

High-resolution (1 × 1 × 1 mm3) T1-weighted structural images were collected, once on every session. The scan had 192 axial slices with a 256 × 256 voxels grid and TR = 1900, TE = 2.92 ms and time to inversion = 900 ms.

### fMRI Data Analysis

Preprocessing Data were analysed using Statistical Parametric Mapping (SPM8, Wellcome Trust Centre for Neuroimaging) in Matlab. All functional and anatomical images from all the sessions were reoriented in the AC-PC orientation. The volumes in every block were realigned to the first volume in the session to correct for participant motion. T1-weighted anatomical image was then co-reregistered to the mean volume of all the sessions (generated in the previous step). No normalisation to the Montreal Neurological Institute (MNI) space or smoothing was applied for MVPA analysis at the individual participant level.

#### Univariate analysis

A general linear model (GLM) was built for each individual participant’s data as implemented in SPM8. Designing the regressors of the GLM with the aim of using the resulting beta parameters in the MVPA framework requires a careful balance between the number of regressors, and hence betas, and quality of the estimated model. Using one regressor per trial would result in more beta parameters, i.e. more samples to train and decode, however this usually results in poor estimates of the parameters. On the other extreme having few regressors will generate more robust estimates, but with very few samples for classification. In this study we piloted with several choices of the regressors and we found that a reasonable balance can be achieved by using one regressor per condition per block, i.e. two trials are combined per regressor. The grey matter tissue probability maps generated by the segmentation processing stage were used as explicit masks to restrict the data analysis to grey matter. For each participant the following univariate contrasts were calculated: three T-tests for Face vs. baseline, Voice vs. baseline and Face-Voice vs. baseline; three F-tests comparing the four identities for each presentation condition.

#### Multivariate analysis

The main GLM design resulted in a dataset with 350.4 ± 64.84 beta images per participant covering the 12 conditions. To decode these samples a whole-brain searchlight paradigm was used^23^. In this approach cross-validated classification is carried out at the level of a small sphere scanning the whole brain. As a result each voxel in the output image contains the average cross-validation accuracy of the sphere centered on it. In order to choose a consistent number of voxels per sphere among all participants different sphere radiuses were tested, between 3 and 8 voxels. For our dataset, a 4-voxel radius (8 mm) produced the most consistent voxel number per sphere (21.74 ± 1.43). Using a relatively smaller sphere ensures the classifier is less likely to over-fit on training samples due to a good balance between the number of samples (observations) and the number of voxels within the sphere (features). The number of samples is large enough compared to their dimensionality so the samples are not too sparse in the feature space^35^. The searchlight analysis covered the whole brain, but a sphere was only considered in the analysis if more than 50% of its voxels fell within the grey matter mask of the subject being analysed, in order to restrict analysis to grey matter across the whole brain in each subject. The number of spheres included in the analysis was (mean ± s.d.) n = 46500 ± 6400. In each sphere the cross-validation analysis only included as features the voxels corresponding to grey matter, the rest being discarded.

Each participant’s data were grouped into training (80% of samples) and testing (the remaining 20%) sets. The training samples were chosen randomly and were then used to train a linear Support Vector Machine (SVM) classifier (as implemented by libSVM) to decode the four identities from the samples in the testing set. This process is then repeated 100 times and the average accuracy value is assigned to the voxel in the centre of the sphere. SVM is usually able to solve only two class problems. In order to extend it to 4-class problems, we used the one-versus-one approach as implemented in libSVM^36^.

#### Empirical Chance Level

In theory the chance level is c = 1/n, where n is the number of classes (c = 0.25 in this study). However, practically the chance level might differ due to the structure of the data or bias in the labelling that affects the training of the classifier. To address this issue an empirical chance level (c’) must be estimated. For each sphere the labels of the training set were shuffled, i.e. samples were assigned wrong labels, before feeding them to the classifier. The classifier was then tested on the test dataset. This step is usually repeated a large number of times (>1000) to estimate the distribution of the chance level per voxel. However, due to the large number of spheres, this was impractical. Here, we only ran the chance level estimation 100 times per sphere as we were only interested in the mean value of the empirical chance level.

#### Significance test

The empirical chance level estimated per sphere was used in conjunction with a two-tailed binomial test^22^,^37^. The resulting p-value maps were thresholded at an FDR q-value of 0.01 to correct for multiple comparisons. The surviving voxels are then clustered, using xjview (http://www.alivelearn.net/xjview), and only clusters with more than 20 voxels were considered in the final accuracy maps.

#### Group Level

We decided to collect large numbers of samples in a small number of participants: this allowed us enough statistical power to perform analyses at the single-participant level. As a consequence of the small number of subjects, we decided not to run random effect analyses at the group level, which would be underpowered, but rather to examine voxel clusters located in consistent locations across participants using coincidence maps: the non-clustered, FDR corrected individual accuracy maps were normalized to MNI space and a voxel was included in the group image if it was significant in at least 4 out of the 5 of participant maps.

This multivariate approach was repeated for four different conditions: (1) Face Classification: only beta images corresponding to face stimuli were classified; (2) Voice Classification: only beta images corresponding to voice stimuli were classified; (3) Face-Voice Cross-Classification: Face-related beta images were used for training while Voice-related beta images were used for testing within the cross-validation paradigm; (4) Voice-Face Cross-Classification: Voice-related beta images were involved in training the classifier while the Face-related beta images were used to test the classifier. The first two conditions were termed “within modality classification” and the last two conditions were termed “cross-modality classification”.

## Additional Information

**How to cite this article**: Awwad Shiekh Hasan, B. *et al*. "Hearing faces and seeing voices": Amodal coding of person identity in the human brain. *Sci. Rep.* **6**, 37494; doi: 10.1038/srep37494 (2016).

**Publisher's note:** Springer Nature remains neutral with regard to jurisdictional claims in published maps and institutional affiliations.

## Supplementary Material

Supplementary Information

## Figures and Tables

**Table 1:** Classification peaks.

Cortical Area	X	Y	Z	Cluster size	Classification
Within-Modality Classification					
Left Fusiform Gyrus	−38	−72	−14	12	Face
Left Posterior Temporal	−44	−64	6	23	Voice
Left STS	−58	−30	−2	35	Voice
Right Inferior Temporal Gyrus	50	−58	−12	43	Face
Right Middle Temporal Gyrus	60	−28	−14	6	Voice
Right Middle Temporal Gyrus	54	−30	−4	7	Voice
Right Posterior STS/STG	52	−64	14	50	Face, Voice
Across-Modality Classification					
Left Inferior Frontal Gyrus	−50	16	6	74	Both directions
Left Inferior Temporal Gyrus	−54	−58	−6	15	Face->Voice
Left Superior Temporal Sulcus	−54	−6	−14	32	Both directions
Left Middle Temporal Gyrus	−54	−34	−2	52	Face->Voice
Left Middle Temporal Gyrus	−58	−58	10	23	Both directions
Right Supramarginal Gyrus	66	−26	28	13	Voice->Face
Right Inferior Temporal Gyrus	48	−68	−8	34	Both directions
Right Inferior Temporal Gyrus	64	−36	−14	19	Voice->Face
Right Middle Temporal Gyrus	54	0	−20	23	Face->Voice
Right Middle Temporal Gyrus	66	−40	4	29	Both directions
Right Superior Temporal Sulcus	64	−24	−4	19	Both directions
Right Superior Temporal Pole	48	16	−18	24	Voice->Face

For each cluster of sphere centers resulting in above-chance (q < 0.01 FDR) classification accuracy in 4 or 5 participants are given approximate cortical area names, MNI coordinates (in mm), cluster size (in voxels) and type of classification.
