# Supplementary material for: “Hearing faces and seeing voices”: Amodal coding of person identity in the human brain
Source: Sci Rep. 2016 Nov 24;6:37494. doi: 10.1038/srep37494 (PMC5121604; doi:10.1038/srep37494)

Supplementary Information

"Hearing faces and seeing voices":

Amodal coding of person identity in the human brain

Bashar Awwad Shiekh Hasan[1,2], Mitchell Valdes-Sosa[3], Joachim Gross[1], Pascal Belin[1,4,5]

Figure S1. Individual cross-classification accuracy maps for the five participants (P1-P5). Voxels are shown if they survive the FDR q<0.01 test. Red voxels: Train classifier on Voice data and test on Face data. Blue voxels: Train classifier on Face and test on Voice. Purple voxels: overlap of red and blue voxels. Only clusters containing more than 20 voxels are shown.

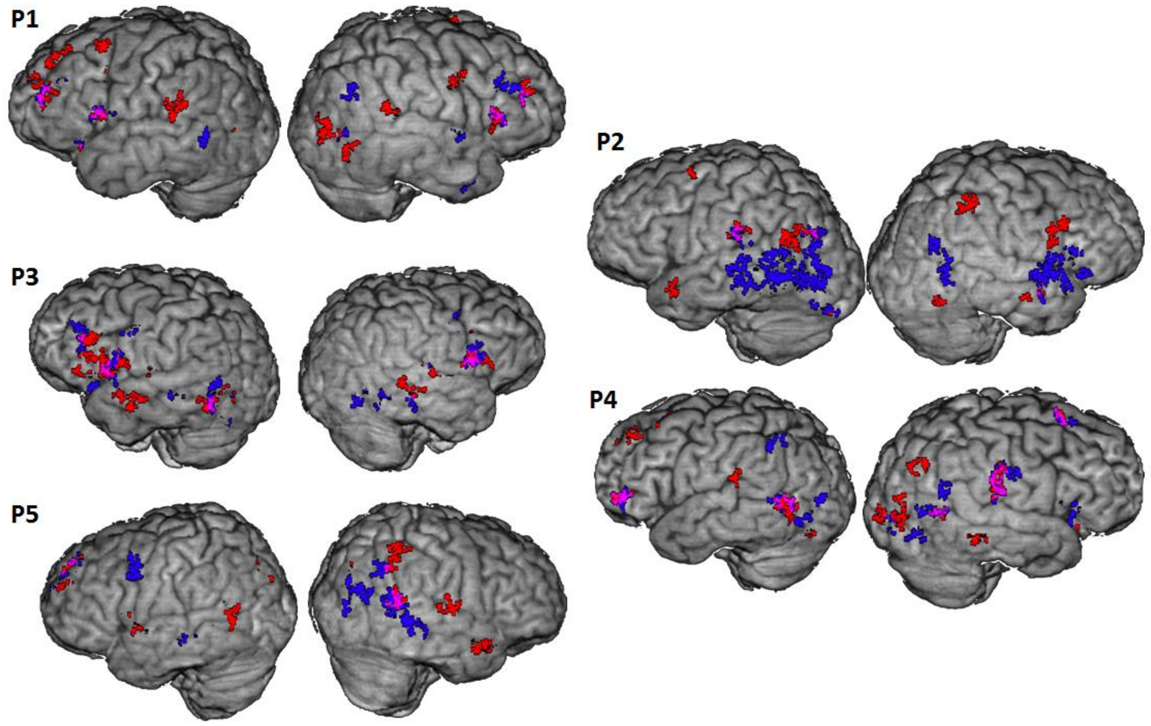

Supplement: Supplementary Information [file srep37494-s1.pdf]
